# Autologous matrix-induced chondrogenesis is effective for focal chondral defects of the knee

**DOI:** 10.1038/s41598-022-13591-6

**Published:** 2022-06-04

**Authors:** Filippo Migliorini, Nicola Maffulli, Alice Baroncini, Andreas Bell, Frank Hildebrand, Hanno Schenker

**Affiliations:** 1 Department of Orthopaedic, Trauma, and Reconstructive Surgery, RWTH University Hospital, Pauwelsstraße 30, 52074 Aachen, Germany; 2 Department of Orthopaedic and Trauma Surgery, Eifelklinik St. Brigida, 52152 Simmerath, Germany; 3 Department of Medicine, Surgery and Dentistry, University of Salerno, 84081 Baronissi, SA Italy; 4 School of Pharmacy and Bioengineering, Keele University Faculty of Medicine, ST4 7QB Stoke on Trent, England; 5 Queen Mary University of London, Barts and the London School of Medicine and Dentistry, Centre for Sports and Exercise Medicine, Mile End Hospital, E1 4DG London, England

**Keywords:** Medical research, Outcomes research, Stem-cell research

## Abstract

Focal chondral defects of the knee are common and their management is challenging. This study investigated the efficacy and safety of Autologous Matrix-Induced Chondrogenesis (AMIC) for focal chondral defects of the knee. A systematic review and meta-analysis was conducted (according to the 2020 PRISMA statement) to investigate the efficacy of AMIC in improving symptoms and to compare AMIC versus microfracture (MFx). In January 2022, the following databases were accessed: Pubmed, Web of Science, Google Scholar, Embase. No time constrain was used for the search. All the clinical trials investigating AMIC and/or those comparing AMIC versus MFx for focal chondral defects of the knee were accessed. Only studies published in peer reviewed journals were considered. Studies which investigated other locations of the defects rather than knee were not eligible, nor those reporting data form mixed locations. Studies which reported data on revision settings, as well as those investigating efficacy on kissing lesions or multiple locations, were not suitable. The mean difference (MD) and odd ratio (OR) effect measure were used for continuous and binary data, respectively. Data from 18 studies (548 patients) were retrieved with a mean follow-up of 39.9 ± 26.5 months. The mean defect size was 3.2 ± 1.0 cm^2^. The visual analogue scale (VAS) decreased of − 3.9/10 (95% confidence interval (CI) − 4.0874 to -3.7126), the Tegner Activity Scale increased of + 0.8/10 (95% CI 0.6595 to 0.9405). The Lysholm Knee Scoring System increased of + 28.9/100 (95% CI 26.8716 to 29.1284), as did the International Knee Documentation Committee (IKDC) + 33.6/100 (95% CI 32.5800 to 34.6200). At last follow-up no patient showed signs of hypertrophy. 4.3% (9 of 210) of patients underwent revision procedures. The rate of failure was 3.8% (9 of 236). Compared to MFx, AMIC demonstrated lower VAS score (MD: − 1.01; 95% CI − 1.97 to 0.05), greater IKDC (MD: 11.80; 95% CI 6.65 to 16.94), and lower rate of revision (OR: 0.16; 95% CI 0.06 to 0.44). AMIC is effective for focal chondral defects of the knee. Furthermore, AMIC evidenced greater IKDC, along with a lower value of VAS and rate of revision compared to MFx.

## Introduction

Focal chondral defects of the knee are common^1,2^. Chondral defects impact negatively sport participation and the quality of life of affected patients^3^. If left untreated, chondral defects have limited chance to heal, and chronic pain may occur^4–6^. The management of chondral defects is challenging with unpredictable results^7,8^. For symptomatic defects smaller than 2 cm^2^, microfractures (MFx) have been proposed^9–12^. MFx is a bone marrow stimulating procedure of simple execution which can be conducted in a fully arthroscopic fashion^13^. During MFx, the cartilage is debrided to its viable border, and microfractures are performed to promote cell migration from the subchondral bone^14,15^. The bone marrow is the major hematopoietic and lymphoid organ, a niche to support self-renewal and differentiation of hematopoietic stem cells (HSC), multipotent progenitors (MPP), and lineage committed progenitors to produce blood cells^16–18^. Subchondral bone marrow cells are believed to enhance cartilage repair^19–21^. However, for bigger defects, the blood clot formed following MFx does not have enough mechanical resistance to remain in situ^22^. To overcome this limitation, in 2005, Behrens et al.^23^ firstly described an enhanced microfractures technique, which developed into Autologous Matrix-Induced Chondrogenesis (AMIC). In AMIC, a resorbable membrane is used to stabilize the clot and keep it stable in the joint cavity^24,25^. Different from other chondral procedures, AMIC does not necessitate to harvest any autologous tissue and is performed in a single session surgery^26,27^. These features make AMIC of special interest to both patients and surgeons^28^.

Several clinical studies evaluating the efficacy and safety of AMIC for focal chondral defects of the knee have been published^24,25,29–37^. However, in the past few years several studies have been published which have not yet been included in previous review^22,27,38–42^. Therefore, a systematic review and meta-analysis was conducted. The primary purpose of the present study was to investigate the efficacy and safety of AMIC for focal chondral defects of the knee. The secondary purpse was to investigate whether AMIC for focal chondral defects of the knee promotes a better outcome than MFx. We hypothesised that AMIC performed in the knee may be effective and safe to manage symptomatic chondral defects.

## Methods

### Eligibility criteria

All the clinical trials investigating AMIC and/or those comparing AMIC versus MFx for focal chondral defects of the knee were accessed. Only studies published in peer reviewed journals were considered. According to the author´ language capabilities, articles in English, German, Italian, French and Spanish were eligible. Only studies with level I to IV of evidence, according to Oxford Centre of Evidence-Based Medicine^43^, were considered. Reviews, opinions, letters, editorials were not considered. Studies which investigated other locations of the defects rather than knee were not eligible, nor were those reporting data from mixed locations. Studies which reported data on revision settings, and those investigating the efficacy of these techniques on kissing lesions or multiple locations, were not eligible. Animals, in vitro, biomechanics, computational, and cadaveric studies were not eligible. Missing quantitative data under the outcomes of interests warranted the exclusion of the study.

### Search strategy

This study was conducted according to the Preferred Reporting Items for Systematic Reviews and Meta-Analyses: the 2020 PRISMA statement^44^. The PICOT algorithm was preliminary pointed out:

- P (Problem): knee chondral defect;
- I (Intervention): AMIC;
- C (Comparison): MFx;
- O (Outcomes): PROMs, rate of hypertrophy, failure, and revision surgery.
- T (Timing): minimum 12 months follow-up.

In January 2022, the following databases were accessed: Pubmed, Web of Science, Google Scholar, Embase. No time constrain was set for the search. The following matrix of keywords were used in each database to accomplish the search: (*knee*) AND (*chondral defects* OR *chondropathy* OR *cartilage defects*) AND (*Autologous Matrix-Induced Chondrogenesis* OR *AMIC* OR *surgery* AND *microfractures*) AND (*pain* OR *symptoms* OR *outcome* AND *patient reported outcome measures* OR PROMs) OR (*complications* AND *revision* AND *hypertrophy* AND *failure*). No additional filters were used in the databases search.

### Selection and data collection

Two authors (F. M. and H. S.) independently performed the database search. All the resulting titles were screened by hand and, if suitable, the abstract was accessed. The full-text of the abstracts which matched the topic were accessed. If the full-text was not accessible or available, the article was not considered for inclusion. A cross reference of the bibliography of the full-text articles was also performed for inclusion. Disagreements were debated and mutually solved by the authors. In case of further disagreements, a third senior author (N. M.) took the final decision.

### Data items

Two authors (F. M. and H. S.) independently performed data extraction. The following data at baseline were extracted: author, year of publication and journal, length of the follow-up, number of patients with related mean age and BMI. Data concerning the following PROMs were collected at baseline and at last follow-up: Visual Analogue Scale (VAS), Tegner Activity Scale^45^, Lysholm Knee Scoring Scale^46^, and International Knee Documentation Committee (IKDC)^47^. The minimum clinically important difference (MCID) for the VAS was 2.7/10, 10/100 for the Lysholm score, 15/100 for the IKDC, 0.5/10 for the Tegner score^48–50^. Data from the following complications were also collected: hypertrophy, failures, and revision surgeries.

### Assessment of the risk of bias and quality of the recommendations

The risk of bias were evaluated in accordance with the guidelines in the Cochrane Handbook for Systematic Reviews of Interventions^51^. Two reviewers (F. M. and H. S.) evaluated the risk of bias of the extracted studies independently. Disagreements were solved by a third senior author (N. M.). Randomised controlled trials (RCTs) were evaluated using the risk of bias of the software Review Manager 5.3 (The Nordic Cochrane Collaboration, Copenhagen). The following endpoints were evaluated: selection, detection, performance, attrition, reporting, and other bias. Non-RCTs were evaluated using the Risk of Bias in Nonrandomised Studies of Interventions (ROBINS-I) tool^52^. The quality of evidence of collective outcomes were evaluated using the Grading of Recommendations, Assessment, Development, and Evaluation (GRADE) system was used^53,54^.

### Synthesis methods

The statistical analyses were performed by the main author (F. M.) following the recommendations of the Cochrane Handbook for Systematic Reviews of Interventions^55^. For descriptive statistics, mean and standard deviation were used. To evaluate the improvement from baseline to last follow-up, the SPSS software was used. The mean difference (MD) was calculated, with 95% confidence interval (CI). The paired t-test was performed with values of P < 0.05 considered statistically significant. To compare AMIC versus MFx, a meta-analysis was conducted using the software Review Manager 5.3 (The Nordic Cochrane Collaboration, Copenhagen). For descriptive statistics, mean difference and standard deviation were used. The T-test was performed to assess baseline comparability, with values of P > 0.1 considered satisfactory. For continuous data, the inverse variance method with mean difference (MD) effect measure was used. For binary data, the Mantel–Haenszel method with odd ratio (OR) effect measure was used. The CI was set at 95% in all the comparison. Heterogeneity was assessed using $\chi$ ^2^ and Higgins-I^2^ tests. If $\chi$ ^2^ > 0.05, no statistically significant heterogeneity was found. A fixed model effect was used as default. If $\chi$ ^2^ < 0.05 and Higgins-I^2^ > 60% high heterogeneity was found and a random model effect was used for analysis. Overall values of P < 0.05 were considered statistically significant.

### Ethical approval

This study complies with ethical standards.

### Registration and protocol

The present review was not registered.

## Results

### Study selection

**Figure 1:**
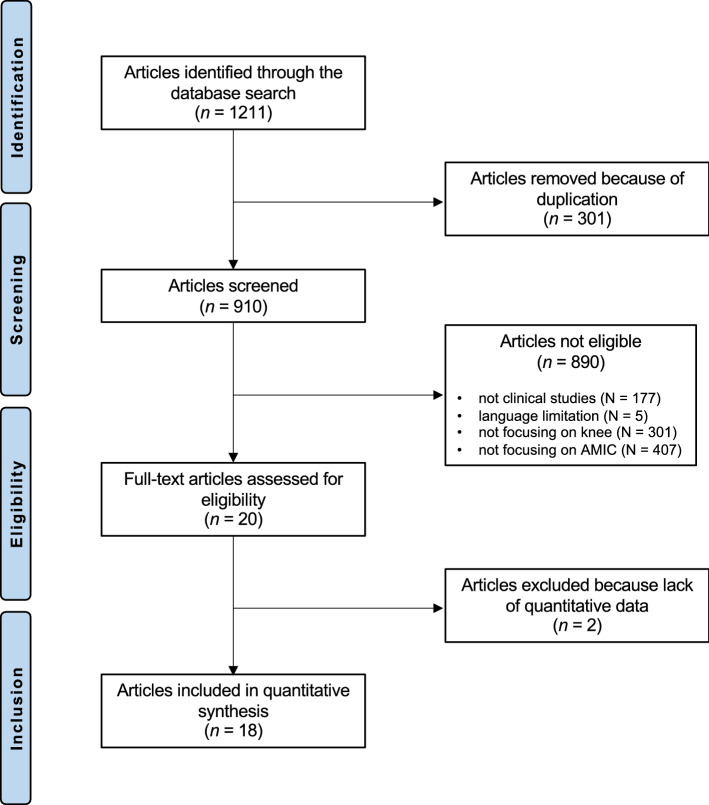
PRISMA flow chart of the literature search.

The literature search resulted in 1211 articles. Of them, 301 were excluded because of duplication. A further 890 studies were excluded as they did not match the eligibility criteria: not clinical studies (N = 177), language limitation (N = 5), not focusing on knee (N = 301), not focusing on AMIC (N = 407). Two studies were not included as they did not report quantitative data under the outcomes of interest. This left 18 studies for inclusion. The results literature search are shown in Fig. 1.

### Risk of bias assessment

**Figure 2:**
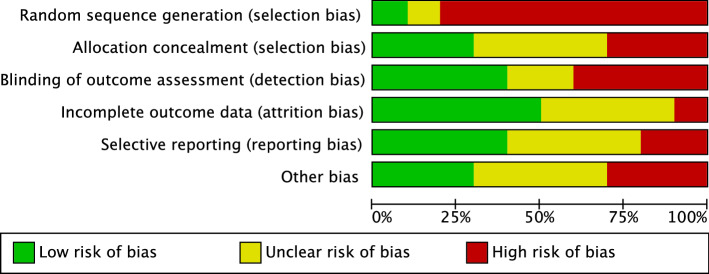
Cochrane risk of bias tool. The risk of selection bias analysed the random sequence generation and the allocation concealment. The risk of detection bias in the blinding procedure during the outcome assessment were analysed. The risk of attrition bias refers to incomplete outcome data, such as missing outcome data from attrition during study enrollment or analysis. The risk of reporting bias refers to the selective publication of results based on their statistical or clinical relevance. If the authors identified additional risk of bias, these were considered as “other bias”. The risk of bias was evaluated in percentage as low, high, or unclear.

The Cochrane risk of bias tool was performed to investigate the risk of bias of RCTs. Given the number of retrospective studies included in the present investigation, the risk of selection bias was moderate. Few authors performed assessor blinding, leading to a moderate risk of detection bias. The risk of attrition and reporting biases was moderate, as was the risk of other bias. Concluding, the risk of bias graph evidenced a moderate quality of the methodological assessment of RCTs (Fig. 2).

The ROBINS-I was applied to investigate the risk of bias of non-RCTs. No study evidenced critical risk of bias. Given the overall acceptable quality of the included studies, the overall risk of bias was moderate (Table 1).

**Table 1:** The ROBINS-I of non-RCTs.

Author, year	Confounding	Participant selection	Classification of interventions	Deviations from intended intervention	Missing data	Measurement of outcomes	Selection of reported results	Overall risk of bias
Astur et al. 2018 ^30^	Low	High	High	Low	Moderate	Low	Low	Moderate
Chung et al. 2014 ^31^	Low	Low	Moderate	Low	Low	Moderate	Low	Moderate
Enea et al. 2013 ^32^	Moderate	Moderate	Low	Moderate	Low	Low	High	Moderate
Enea et al. 2015 ^33^	Low	Low	Moderate	Low	High	High	Low	Moderate
Gille et al. 2013 ^34^	Low	High	High	Moderate	High	Low	High	Moderate
Gille et al. 2020 ^38^	Moderate	Low	High	Low	Low	High	Low	Moderate
Gudas et al. 2018 ^35^	Low	Moderate	Low	Moderate	Low	Moderate	Moderate	Moderate
Lahner et al. 2018 ^36^	Low	Low	Low	Low	High	Low	Low	Moderate
Migliorini et al. 2021 ^39^	Moderate	High	High	Low	Low	High	Low	Moderate
Migliorini et al. 2021 ^40^	Moderate	High	High	Moderate	Moderate	Low	Moderate	Moderate
Miyahira et al. 2020 ^22^	Low	Low	Low	High	High	Low	Low	Moderate
Schagemann et al. 2018 ^25^	Moderate	Low	High	Low	Moderate	High	Low	Moderate
Schiavone Panni et al. 2018 ^24^	Low	Moderate	Moderate	Low	Moderate	Low	High	Moderate
Tradati et al. 2020 ^41^	Moderate	Moderate	Low	Moderate	Low	Low	Moderate	Moderate
Waltenspül et al. 2021 ^42^	Low	Low	Moderate	Low	High	Moderate	Low	Moderate

### Study characteristics and results of individual studies

Data from 548 patients were retrieved. 33% (180 of 548 patients) were female. The mean follow-up was 39.9 ± 26.5 months. The mean age was 27.0 ± 5.9 years and the mean BMI 27.1 ± 1.3 kg/m^2^. The mean defect size was 3.2 ± 1.0 cm^2^. The generalities and demographic of the included studies is shown in Table 2.

**Table 2:** Generalities and patient baseline of the included studies (RCT: randomised controlled trial).

Author, year	Journal	Desgin	Patients (*n*)	Follow up (*months*)	Female (*%*)	Mean age	Mean BMI	Defect size (*cm*^*2*^)
Anders et al. 2013 ^29^	*Open Orthop J*	RCT	8	24.0	12%	35.0	27.4	3.8
			13		23%	39.0	27.7	3.8
Astur et al. 2018 ^30^	*Rev Bras Orthop*	Non-RCT	7	12.0	14%	37.2		2.1
Chung et al. 2014 ^31^	*Knee Surg Sports Traumatol Arthrosc*	Non-RCT	24		42%	47.4		1.3
De Girolamo et al. 2019 ^27^	*J Clin Med*	RCT	12	100.0	38%	30.0		3.8
			12		50%	30.0		3.4
Enea et al. 2013 ^32^	*Knee*	Non-RCT	9	22.0	45%	48.0		2.6
Enea et al. 2015 ^33^	*Knee*	Non-RCT	9	29.0	44%	43.0		2.5
Gille et al. 2013 ^34^	*Arch Orthop Trauma Surg*	Non-RCT	57	24.0	33%	37.0		3.4
Gille et al. 2020 ^38^	*Orthop J Sports Med*	Non-RCT	131	12.0	37%	36.6	25.7	3.3
Gudas et al. 2018 ^35^	*J Orthop Surg*	Non-RCT	15	54.0	33%	31.0		5.3
Lahner et al. 2018 ^36^	*Biomed Res Int*	Non-RCT	9	14.7		48.0	29.3	2.1
Migliorini et al. 2021 ^39^	*LIFE*	Non-RCT	52	43.7	35%	29.5	27.1	2.8
Migliorini et al. 2021 ^40^	*LIFE*	Non-RCT	27	45.1	48%	35.8	26.9	2.7
Miyahira et al. 2020 ^22^	*Rev Bras Ortop*	Non-RCT	15	12.0	20%	39.2	27.6	1.6
Schagemann et al. 2018 ^25^	*Arch Orthop Trauma Surg*	Non-RCT	20	24.0	35%	38.0	27.0	3.1
			30		43%	34.0	23.9	3.4
Schiavone Panni et al. 2018 ^24^	*Knee Surg Sports Traumatol Arthrosc*	Non-RCT	21	84.0				
Tradati et al. 2020 ^41^	*J Clin Med*	Non-RCT	14	68.2	36%	38.4		4.5
Volz et al. 2017 ^37^	*Int Orthop*	RCT	17	60.0	29%	34.0	27.4	3.8
			17		11%	39.0	27.6	3.9
Waltenspül et al. 2021 ^42^	*Cartilage*	Non-RCT	29	49.2		27.9	27.6	3.9

### Efficacy of AMIC

The VAS decreased of – 3.9/10 (95% CI − 4.0874 to − 3.7126), the Tegner Activity Scale increased of + 0.8/10 (95% CI 0.6595 to 0.9405). The Lysholm Knee Scoring System increased of + 28.9/100 (95% CI 26.8716 to 29.1284), as did the IKDC + 33.6/100 (95% CI 32.5800 to 34.6200). These results are shown in greater detail in Table 3.

**Table 3:** Improvements in PROMs from baseline to the last follow-up (FU: follow-up; MD: mean difference; CI: confidence interval; IKDC: International Knee Document Committee).

Endpoint	Baseline	Last FU	MD	95%CI	P
Visual analogue scale	6.5 ± 1.0	2.6 ± 2.0	− 3.9	− 4.0874 to − 3.7126	0.0001
Tegner activity scale	3.7 ± 1.6	4.5 ± 0.5	0.8	0.6595 to 0.9405	0.03
Lysholm knee scoring system	53.7 ± 11.5	81.7 ± 7.0	28.0	26.8716 to 29.1284	< 0.0001
IKDC	46.1 ± 8.9	79.7 ± 8.3	33.6	32.5800 to 34.6200	< 0.0001

### Complications

At last follow-up, no patient showed signs of hypertrophy. 4.3% (9 of 210) of patients underwent revision surgery. The rate of failure was 3.8% (9 of 236).

### AMIC compared to MFx

**Figure 3:**
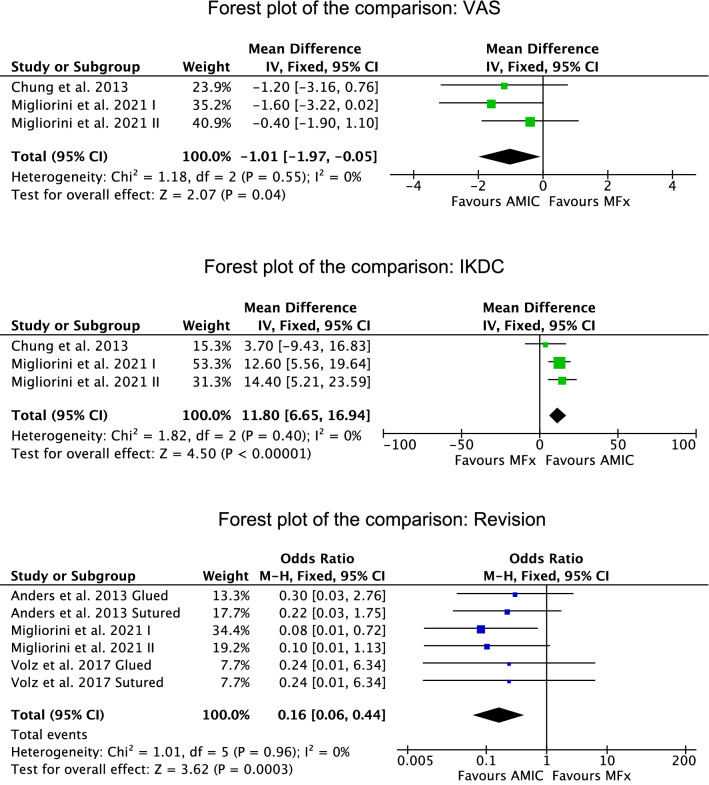
Meta-analyses: forest plot of each comparison (*IV* inverse variance, *OR* odd ratio, *MD* mean difference, *MH* Mantel–Haenszel, *CI* confidence interval). The final effect and the relative confidence interval are represented respectively by the diamond and its lateral ends. The vertical line indicates the no effect threshold. The effect and the respective confidence interval of each study are represented by the square and the horizontal line, respectively.

Five studies were included in the meta-analyses^29,31,37,39,40^. At a mean follow-up of 40.3 months, the AMIC group demonstrated lower VAS score (MD: − 1.01; 95% CI − 1.97 to 0.05) and greater IKDC (MD: 11.80; 95% CI 6.65 to 16.94). At a mean follow-up of 43.6 months, the AMIC group demonstrated lower rate of revision (OR: 0.16; 95% CI 0.06 to 0.44). These results are shown in greater detail in Fig. 3.

### Quality of the recommendations

**Figure 4:**
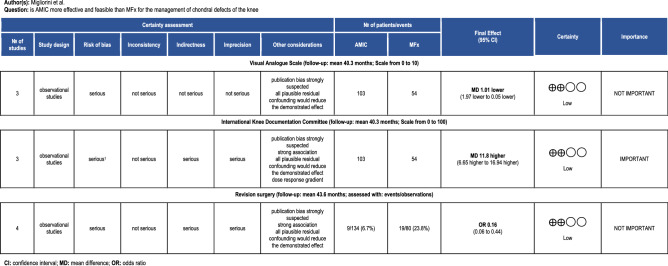
The overall quality of evidence of collective outcomes according to the GRADE approach was low.

The GRADE found limited effect in the estimated effect, and the true effect might be substantially different from the estimated effects. This relates to a low quality of the recommendations outcome rate of revision, and in IKDC and VAS scores (Fig. 4).

## Discussion

### General interpretation and clinical implication

The management of chondral defects of the knee is controversial, with unpredictable results. To date, no modality is considered definitive, and residual defect and symptoms recurrence is common. According to the main findings of the present study, AMIC seems to be effective to manage focal chondral defects of the knee. The increase in PROMs were greater than their MCID^48–50^. Furthermore, AMIC evidenced greater IKDC values, along with a lower value of VAS and lower rate of revision compared to MFx. Differently to other chondral procedures, AMIC does not necessitate to harvest or expand any autologous tissue, and is performed in a single session surgery. Therefore, AMIC should be considered in selected patients with symptomatic chondral defect of the knee.

Previous systematic reviews evaluated the efficacy of AMIC. Gao et al.^56^ evaluated the efficacy of AMIC in the knee including 12 studies. They found reduction in VAS and improvement of the Lysholm score within the first two years follow-up, but no improvement from two to five years follow-up^56^. Steinwachs et al.^57^ also performed a systematic review including 12 studies on AMIC. The Lysholm score, IKDC, and VAS were improved within the first two years follow-up alike, but they continued to improve after 3 years^57^. Previous systematic reviews also compared AMIC versus other common surgical strategies for chondral regeneration. Kim et al.^58^ compared AMIC (13 studies) versus MFx (18 studies). They evidenced greater values at IKDC evaluation compared to MFx, with no difference in Lysholm score, Tegner activity scale, and VAS for pain^58^. A recent systematic review of the same study group compared AMIC to matrix-induced autologous chondrocyte implantation (mACI) on the knee^59^. Although there were not statistical differences between the two interventions, given the single step procedure, avoidance of autologous cartilage harvest, and the need for chondrocyte expansion in a separate laboratory setting, AMIC may be preferable to mACI^59^. Another recent systematic review compared AMIC versus other chondral procedures including only RCTs^60^. Overall, AMIC demonstrated efficacy and safety in small- to medium-sized cartilage defects of the knee^60^.

### Limitations of the included evidence

Between studies variability was evident. Most authors used a resorbable collagen I/III porcine derived membrane (Chondroguide®, Geistlich Biomaterials, Wolhusen, Switzerland)^22,24,25,27,29–31,34–36,38–40^. Enea et al.^32^ in 2013 published on the clinical application of AMIC using a polyglycolic acid and hyaluronic acid membrane enhanced with bone marrow concentrate. The same study group in 2015 published the results of AMIC using Biocollagen MeRG® collagen membrane (Bioteck, Vicenza, Italy) enhanced with bone marrow concentrate. Variability was also detected in the membrane fixation technique. Most authors fixed the membrane using fibrin glue^22,24,25,27,31–36,38–41^. In addition to its sealing, haemostatic, and adhesive proprieties, fibrin glue supports chondrocytes migration and proliferation^61–70^. Moreover, fibrin glue stimulates osteochondral scaffold fixation and cartilage regeneration^71–74^. Two authors compared AMIC fixed using fibrin glue versus suture^29,37^. Both authors reported better outcomes in the glued AMIC group. Membrane sutures produces fissures in the articular cartilage which may not heal, and may enlarge with time^75,76^. Suturing induces local cartilage impairment which may lead to pain, reduced healing, and premature osteoarthritis^77^. Most authors performed AMIC using a mini-arthrotomy or an arthrotomy^22,27,29,31,34–41^. Some authors^24,25,32,33^ used an arthroscopic technique to perform AMIC. These between studies variabilities may increase the risk of publication bias, and reduce the reliability of the present study.

### Limitations of the review

The retrospective design of 55% (10 of 18) of the included studies represents another important limitation of the present investigation. Given the limited data available for inclusion, randomised and non randomised studies were not analysed separately. Most authors mixed patients who underwent chondral procedures on the femorotibial and patellofemoral joints, without reporting results separately. Moreover, most authors reported data from patients who underwent combined procedures. The description of the surgical approach, diagnosis, and rehabilitation protocols were often adequate, as were the criteria selection, outcome measures, and related timing of assessment. General health measures were seldom described, and the procedure to assess outcomes were often biased. To ensure the high quality of the included research and related validity of the findings, grey literature and not-peer reviewed articles were not considered. This may limit the number of investigations for inclusion and may limit the strength of the present study. The histopathology of the newly formed cartilage was not compared in the present meta-analysis. The characteristic of the new-formed cartilage at Magnetic Resonance Imaging (MRI) sequences were not investigated. Several studies analysed the magnetic resonance observation of cartilage repair tissue (MOCART) scoring system to evaluate the quality of the chondral regeneration. However, the MOCART score demonstrated no association with patient characteristics and with the surgical outcome in patients who underwent surgical management for knee and talus chondral defects^78^. The reviewers (F. M. and H. S.) who performed the literature search, data extraction, risk of bias assessment were the main authors of two of the included studies^39,40^. This may generate conflicts. Finally, a duplicate process in the literature search and data extraction was not conducted. These limitations impacted negatively on the reliability of the present study. Therefore, results from the present systematic review and meta-analysis should be considered carefully.

## Conclusion

AMIC seems to be effective for the management of focal chondral defects of the knee. Furthermore, AMIC evidenced greater IKDC score, along with a lower value of VAS and rate of revision compared to MFx. The limited quantity and quality of the included studies limit the reliability of the present results and should be interpreted within the limitation of the present study.

## Supplementary Information

Supplementary Information.

## Data Availability

The datasets generated during and/or analysed during the current study are available throughout the manuscript.
